# Predictive value of Glycosylated Fibronectin (GlyFn)/Placenta Growth Factor (PlGF) ratio for high-risk pregnancies: a cohort study

**DOI:** 10.1186/s12884-025-07853-0

**Published:** 2025-07-17

**Authors:** Wahyudi Wirawan, Sofie Rifayani Krisnadi, Anita Deborah Anwar, Annisa Dewi Nugrahani

**Affiliations:** 1 Department of Obstetrics and Gynecology, Faculty of Medicine, Fetomaternal Division, Universitas Padjadjaran– Dr. Hasan Sadikin General Hospital Bandung, Bandung, West Java Indonesia; 2 Doctoral Programme, Faculty of Medicine, Universitas Padjadjaran, Bandung, West Java Indonesia

**Keywords:** Angiogenic, Glycosylated fibronectin, Preeclampsia, Placenta growth factor

## Abstract

**Background:**

Preeclampsia (PE) is a hypertensive disorder in pregnancy involving multiple organ systems, primarily due to impaired placental development during the first trimester. Reduced Placenta Growth Factor (PlGF) and elevated Glycosylated Fibronectin (GlyFn) levels reflect these pathological changes. Combining these biomarkers into a ratio may enhance PE prediction in high-risk pregnancies.

**Objective:**

To evaluate the GlyFn/PlGF ratio measured at 20–28 weeks of gestation as a predictor of preeclampsia in high-risk pregnancies across four hospitals in West Java, Indonesia, from April–October 2024.

**Methods:**

This prospective cohort study involved 54 high-risk pregnant women. The GlyFn/PlGF ratio was analyzed at 20–28 weeks to assess predictive accuracy for PE.

**Results:**

Significant differences were noted between PE and non-PE groups in pre-pregnancy weight (65.54 ± 12.69 vs. 58.53 ± 7.85 kg), BMI (45.52 ± 53.73 vs. 19.84 ± 2.97), and systolic blood pressure (153.50 ± 19.42 vs. 100.80 ± 15.38 mmHg) (*P* > 0.05). Risk factors such as prior PE (*P* = 0.01), diabetes mellitus (*P* = 0.03), and gestational hypertension (*P* = 0.02) were more frequent in the PE group. A GlyFn cutoff of 34.31 ng/mL showed a 2.5-fold increased PE risk (sensitivity: 83.33%, specificity: 66.67%, AUC: 0.8071). A PlGF cutoff of 26.72 pg/mL was associated with a 6.8-fold increased risk (sensitivity: 72.22%, specificity: 97.22%, AUC: 0.9147). The GlyFn/PlGF ratio cutoff of 1.288 indicated a 28-fold higher PE risk (sensitivity: 77.78%, specificity: 97.37%, AUC: 0.9244).

**Conclusion:**

The GlyFn/PlGF ratio outperforms individual biomarkers in predicting preeclampsia in high-risk pregnancies, offering a valuable tool for early risk identification and timely intervention.

## Introduction

According to the American College of Obstetricians and Gynecologists (ACOG) Practice Bulletin, preeclampsia (PE) is an early-onset hypertensive condition characterized by elevated blood pressure ≥ 140/90 mmHg after 20 weeks of gestation, accompanied by qualitative proteinuria (+ 1 or > 0.3 g/24 hours) or signs of maternal end-organ dysfunction, including renal insufficiency, liver involvement, neurological or hematological complications [1, 2]. Globally, PE affects approximately 2–8% of pregnancies and contributes to over 70,000 maternal deaths and 500,000 fetal deaths annually. In 2016, seven referral hospitals in Indonesia reported 1,232 cases of PE, with a maternal mortality rate of 2.2%.^3,4^ Data from Dr. Hasan Sadikin General Hospital in Bandung revealed that preeclampsia accounted for 4.0–10.4% of cases, and eclampsia 2.3–4.3%, between 2008 and 2010, contributing to 10.4% of maternal deaths in the hospital [1–5]. 

The exact etiology of preeclampsia remains unclear [4–6]. The condition is associated with high-risk pregnancy factors, including maternal medical history (chronic hypertension, diabetes, renal disease, autoimmune disorders), maternal characteristics (age < 18 or > 40, obesity, prior PE, long interpregnancy intervals, in vitro fertilization/IVF pregnancies), and genetic predisposition [5–8]. These risk factors align with a disrupted pathogenesis, involving impaired uteroplacental circulation, syncytiotrophoblast dysfunction, and angiogenic imbalance [4, 8]. Poor trophoblastic invasion and spiral artery remodeling in early pregnancy result in narrowed arteries, reduced perfusion, placental hypoxia, and ischemia [4]. These triggers decreased angiogenic factors, such as Placenta Growth Factor (PlGF), and increased antiangiogenic factors like soluble FMS-like tyrosine kinase-1 (sFlt-1), disrupting endothelial integrity, contributing to the clinical manifestations of PE, including proteinuria, hypertension, and end-organ damage [8, 9]. 

PlGF serves as a valuable biomarker for preeclampsia, rising between 16 and 18 weeks of gestation and peaking at 29–32 weeks [10]. A pathological decline in PlGF levels indicates syncytiotrophoblast stress caused by hypoxia, inflammation, or oxidative stress, as seen in PE [11, 12]. Combining PlGF with maternal plasma fibronectin (Fn) has been shown to improve PE screening, particularly in populations with lower detection rates [13]. Glycosylated Fibronectin (GlyFn), a complex formed by plasma and cellular Fn [13, 14] has shown strong correlations with endothelial dysfunction and vascular remodeling disorders associated with PE [10–17]. Research highlights the significance of the GlyFn/PlGF ratio as a predictive biomarker for PE in high-risk pregnancies between 20 and 28 weeks of gestation, a critical period for placental and maternal physiological changes [10–12, 15–17]. This ratio captures the elevated GlyFn and reduced PlGF levels characteristic of PE, aiding in risk stratification and antenatal management. Although the sFlt1/PlGF ratio is well established as a predictive biomarker for preeclampsia, its availability and cost can limit its widespread use, particularly in low-resource settings. Additionally, GlyFn has emerged as a promising biomarker associated with endothelial dysfunction and vascular remodeling abnormalities in preeclampsia. Combining GlyFn with PlGF into a ratio may offer an alternative or complementary method for early prediction, potentially enhancing sensitivity and specificity in high-risk populations This study explores the predictive value of the GlyFn/PlGF ratio as a biomarker for PE, focusing on its clinical utility during the critical window of 20–28 weeks of gestation.

## Methods

### Study design and eligibility

This study was a prospective cohort study conducted in four hospitals in West Java, Indonesia, between April and October 2024. A total of 54 high-risk pregnant women were recruited and followed from 20 to 28 weeks of gestation to assess the predictive accuracy of the GlyFn/PlGF ratio for preeclampsia. Participants were selected based on high-risk factors for preeclampsia, including maternal age < 18 or > 40 years, interpregnancy intervals ≥ 10 years, and other established clinical risk factors including obesity (BMI > 30 kg/m²), previous PE history, and conditions such as chronic hypertension, gestational hypertension, diabetes (type 1 and 2), renal disease, autoimmune disorders (e.g., systemic lupus erythematosus and antiphospholipid syndrome), multifetal pregnancies, and pregnancies achieved through assisted reproductive technology or in vitro fertilization (IVF). Additionally, a family history of PE is considered a significant inclusion factor. Eligible participants were identified during routine antenatal care visits at the participating hospitals. Obstetricians screened patients based on their clinical history and risk factors, and eligible women who agreed to participate were enrolled after providing informed consent.

Exclusion criteria included pregnancies complicated by chromosomal abnormalities, fetal malformations, miscarriages, premature rupture of membranes (PROM), or suspected systemic or intraamniotic infections.Dropout criteria included: (1) Withdrawal of consent at any time during the study, (2) loss to follow-up before 28 weeks of gestation, (3) development of any unrelated medical condition necessitating discontinuation from the study.

#### Ethical statement

Ethical approval was obtained from the institutional review board of Hasan Sadikin General Hospital (Approval No: DP.04.03/D.XIV.6.5/125/2024 and informed consent was secured from all participants.

### Sampling and laboratory procedures

The study outlined rigorous procedures for blood sample collection and processing to ensure biomarker accuracy, conducted at Clinical Pathology Laboratory, Hasan Sadikin General Hospital. Blood samples were drawn from the cubital vein using a 5 mL syringe and stored in sterile Vacutainer tubes labeled with patient barcodes. Samples were allowed to coagulate at room temperature for approximately 30 min, then refrigerated at 2–8 °C. Transportation from participating hospitals to the central research laboratory was performed using chiller boxes with dry ice to maintain stable temperatures. Samples were centrifuged at 2,000–3,000 rpm for 20 min to separate serum from sediment. The serum was aliquoted into numbered containers for immunological analysis. Specific reagents, including the Human Glycosylated Fibronectin (GlyFn) ELISA Kit and the Human Placental Growth Factor (PlGF) ELISA Kit, were utilized for biomarker evaluation. The processed biomarker data were meticulously recorded and provided to researchers for further analysis, ensuring the study’s validity and contribution to preeclampsia risk prediction.

### Statistical analysis

The analysis conducted had to align with the type of research problem and the data utilized. For numerical data, normality testing was performed prior to statistical analysis using the Kolmogorov-Smirnov test when the dataset exceeded 50 samples. This test determined whether the data followed a normal or non-normal distribution. Subsequently, statistical analysis was carried out based on the research objectives and hypotheses. A significance test was used to compare the characteristics of two study groups; an independent t-test was applied if the data were normally distributed, while the Mann-Whitney test was employed as an alternative for non-normally distributed data. For categorical data, statistical analysis utilized the Chi-square test when its assumptions were met. If the assumptions were violated, Fisher’s Exact test was used for 2 × 2 tables, and the Kolmogorov-Smirnov test was applied for tables larger than 2 × 2. The Chi-square requirements mandated that no more than 20% of the table’s expected values were below 5. The significance criterion applied was the *p*-value, where *p* ≤ 0.05 indicated statistical significance, and *p* > 0.05 indicated non-significance. Statistical analysis included sensitivity, specificity, PPV, and NPV calculations, along with cut-off point determination using receiving operator characteristics (ROC) curve. The collected data were recorded on specific forms and processed using GraphPad Prism 10 for Mac. A priori power analysis was not performed due to limited available data at the study’s initiation; this is recognized as a limitation.

## Results

### Clinical characteristics of study subjects

Table 1 summarizes the clinical characteristics of the study participants, highlighting key demographic and medical factors relevant to preeclampsia risk.

**Table 1:** Population characteristic

Variables	Value
Maternal Age in Years (Mean, Min-Max)	32.67 (17–44)
Gestational Age in Weeks (Mean, Min-Max)	25.60 (20–33)
Body Weight before Pregnancy in Kilograms/kg (Mean, Min-Max)	63.25 (41–107)
Body Weight Increase during Pregnancy (kg) (Mean, Min-Max)	8.26 (5–22)
Body Mass Index/BMI in kg/m^2^ (Mean, Min-Max)	36.96 (16–37)
Systolic Blood Pressure in mmHg (Mean, Min-Max)	139.3 (100–175)
Diastolic Blood Pressure in mmHg (Mean, Min-Max)	90.10 (56–115)
Previous PE history (n,%)	15 (27.78%)
Diabetes mellitus (n,%)	17 (31.48%)
Gestational Hypertension (n,%)	18 (33.33%)
Pregnancy Interval > 10 years (n,%)	2 (3.70%)
Pregnancy with Assisted Reproductive Technology (n,%)	1 (1.85%)
Chronic Hypertension (n,%)	19 (35.18%)
Autoimmune (Systemic Lupus Erythematosus) (n,%)	5 (9.25%)
Multifetal Pregnancies (n,%)	5 (9.25%)
Renal Disease (n,%)	1 (1.85%)
Genetic History of Preeclampsia/PE (n,%)	14 (25.93%)

This study initially recruited 60 pregnant women suspected of having preeclampsia. After applying the inclusion and exclusion criteria and accounting for a 10% dropout rate (6 subjects: 2 lost to follow-up, 2 withdrew consent, and 2 had incomplete data), a total of 54 subjects were included in the final analysis. These consisted of 36 cases in the preeclampsia group and 18 in the non-preeclampsia group, as detailed in Table 1. As shown in Tables 1 and 36 were categorized into the preeclampsia group and 18 into the non-preeclampsia group. The distribution of maternal demographics and clinical risk factors such as BMI, blood pressure, history of hypertension or preeclampsia, and comorbidities provides context for the population characteristics, with several high-risk factors commonly observed among those included.

These data highlight the distribution of demographic and clinical characteristics that reflect significant maternal risk factors during pregnancy. This analysis underscores the importance of targeted strategies to prevent and manage pregnancy complications, particularly in high-risk populations. The clinical characteristics, maternal medical history, and serum biomarker profiles of the subjects are presented in Table 2.

**Table 2:** Comparison of maternal profiles between PE and non-PE groups

Variables	Total (*n* = 54)		*P*-Value	
	Preeclampsia (PE) (*n* = 36)	Non-Preeclampsia (Non-PE) (*n* = 18)		Effect Estimate (RR/LR, CI 95%)
Clinical Subject Characteristics				
Maternal Age (years, mean ± SD)	33.83 ± 6.20	30.33 ± 7.89	0.08^a^	
Gestational Age (weeks, mean ± SD)	25.53 ± 5.76	25.74 ± 2.96	0.83^a^	
Body Weight before Pregnancy (kg, mean ± SD)	65.54 ± 12.69	58.53 ± 7.85	0.04^a^	
Body Weight Increase during Pregnancy (kg, mean ± SD)	7.788 ± 11.37	5.731 ± 17.14	0.65^a^	
BMI (kg/m^2^, min-max)	45.52 ± 3.73	19.84 ± 2.97	0.04^a^	
SBP (mmHg, mean ± SD)	153.50 ± 19.42	100.80 ± 15.38	0.01^a^	
DBP (mmHg, mean ± SD)	92.78 ± 12.65	77.17 ± 9.50	0.10^a^	
Maternal Medical History				
Previous preeclampsia (PE) history (n,%)	14 (38.5%)	1 (5.5%)	0.01^c^	0.01^c^
Diabetes mellitus (n,%)	15 (41.7%)	2 (11%)	0.03^c^	1.65 (1.17–2.31)
Gestational Hypertension (n,%)	15 (41.7%)	3 (16.7%)	0.02^c^	1.55 (1.08–2.21)
Pregnancy Interval > 10 years (n,%)	0	2 (11%)	0.10^c^	1.57 (1.09–2.31)
Pregnancy with Assisted Reproductive Technology (n,%)	0	1 (5.5%)	0.33^c^	-
Chronic Hypertension (n,%)	14 (38.5%)	5 (27.5%)	0.55^c^	-
Autoimmune (Systemic Lupus Erythematosus) (n,%)	4 (11%)	1 (5.5%)	0.65^c^	1.17 (0.77–1.7)
Multifetal Pregnancies (n,%)	4 (11%)	1 (5.5%)	0.65^c^	1.23 (0.56–1.71)
Renal Disease (n,%)	1 (2.75%)	0	0.99^c^	1.23 (0.56–1.71)
Genetic History of Preeclampsia/PE (n,%)	12 (33%)	2 (11%)	0.10^c^	1.51 (0.31–7.35)
Serum Biomarker Profiles				
GlyFn (ng/mL) (Mean ± SD)	37.6 ± 10.45	27.29 ± 15.43	0.01^a^	2.5
PlGF (pg/mL) (Mean ± SD)	16.11 ± 5.4	42.37 ± 18.08	< 0.01^a^	29.47
Ratio of GlyFn/PlGF	72.52 (1.28–2526)	1.16 (0.37–7.50)	< 0.01^b^	34

If the data followed a normal distribution, it was described using Mean ± SD and analyzed using the independent t-test (a). If not, the data was presented as Min-Max and analyzed with the Mann-Whitney test (b). Categorical data with a sample size < 20 was evaluated using Fisher’s Exact test (c). A *P*-value < 0.05 was considered statistically significant (CI = 95%). Categorical data with a sample size < 20 was analyzed using Fisher’s Exact test (c). A *P*-value < 0.05 was considered statistically significant (CI = 95%)

*BMI* Body Mass Index, *PE* Preeclampsia, *SBP* Systolic Blood Pressure, *DBP* Diastolic Blood Pressure, *SLE* Systemic Lupus Erythematosus, *ART* Assisted Reproductive Technology, *RR* Relative Risk, *LR* Likelihood Ratio, *CI* Confidence Interval

Regarding maternal medical history, factors such as interpregnancy intervals > 10 years, pregnancies achieved through assisted reproductive technology (ART), chronic hypertension, autoimmune disorders (SLE, APS), multifetal pregnancies, kidney disease, and a genetic history of preeclampsia did not show significant differences. However, patients with preeclampsia were more likely to have a history of preeclampsia (RR: *P* = 0.01), diabetes mellitus (RR: *P* = 0.03), and gestational hypertension (RR: *P* = 0.02) compared to the Non-PE group (Table 3). This data shows that most of the subject characteristics were homogeneous between the two study groups. Most maternal risk factors did not significantly differ between the two groups, and thus these factors did not act as confounders in this study.

**Table 3:** Diagnostic performa of serum biomarkers

**Parameter**	***Cut-off Value***	**Sensitivity (%)**	**Specificity (%)**	**PPV (%)**	**NPV (%)**	**AUC** **(95% CI)**
**Biomarker**						
GlyFn	34.31 ng/mL	83.33	66.67	16.67	72.20	0.8071
PlGF	26.71 pg/mL	84.21	97.14	47.97	52.59	0.9045
GlyFn/PlGF Ratio	1.290	94.44	97.22	50.82	49.71	0.9460

Notes: *AUC* Area Under Curve, *NPV* Negative Predictive Value, *PPV* Positive Predictive Value

### Serum biomarker profiles

GlyFn levels were significantly higher in the PE group compared to the Non-PE group (37.36 ± 10.45 vs. 27.29 ± 15.43 ng/mL, *P* = 0.01). Conversely, PlGF levels were significantly lower in the PE group compared to the Non-PE group (16.11 ± 5.4 vs. 42.37 ± 18.08 pg/mL, *P* < 0.01). The GlyFn/PlGF ratio was markedly higher in the PE group than in the Non-PE group (72.52 [1.28–2526] vs. 1.16 [0.37–7.50], *P* < 0.01).

**Fig. 1:**
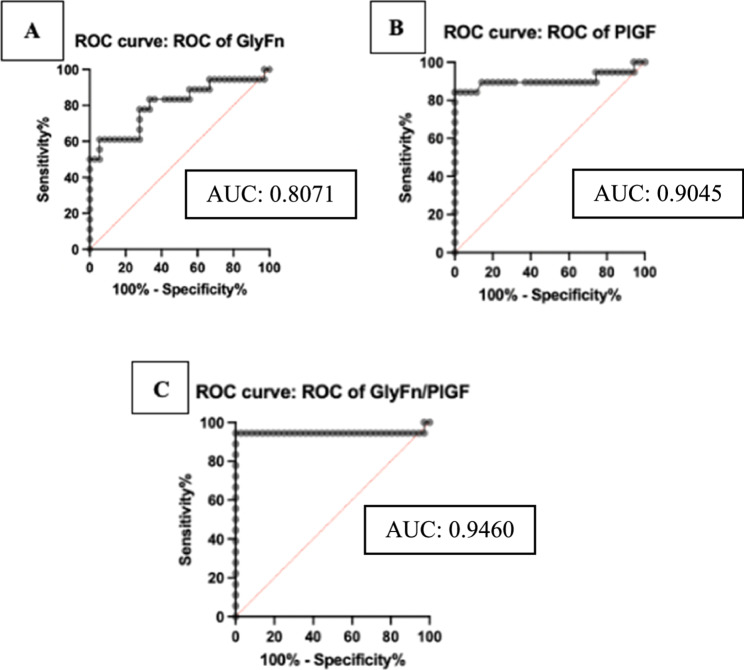
Receiver Operating Characteristic (ROC) analysis of serum biomarkers GlyFn, PlGF, and the GlyFn/PlGF ratio in a prospective cohort study for predicting preeclampsia (PE)

In this study, the GlyFn level had a cutoff value of 34.31 ng/mL. High-risk pregnant women with GlyFn levels exceeding 34.31 ng/mL were found to have a 2.5-fold increased risk of developing preeclampsia (PE), with a sensitivity of 83.33% (95% CI: 29.03%–70.97%) and a specificity of 66.67%. The Positive Predictive Value (PPV) was 16.67%, and the Negative Predictive Value (NPV) was 72.20%. The Likelihood Ratio (LR) was 2.5 (95% CI: 56.01%–84.15%), and the Area Under the Curve (AUC) was 0.8071 (95% CI: 0.6694–0.9448), indicating very good predictive strength (Figure 1A and Table 3).

The PlGF level in this study had a cutoff value of 26.71 pg/mL. High-risk pregnant women with PlGF levels below 26.71 pg/mL had a 6.8-fold increased risk of PE, with a sensitivity of 84.21% (95% CI: 62.43%–94.48%) and a specificity of 97.14% (95% CI: 85.47%–99.85%). The PPV was 47.97%, and the NPV was 52.59%. The LR was 29.47 (95% CI: 71.34%–93.92%), and the AUC was 0.9045 (95% CI: 0.7865–1.000), also indicating very strong predictive strength (Figure 1B and Table 3).

The GlyFn/PlGF ratio showed a cutoff value of 1.290. High-risk pregnant women with a GlyFn/PlGF ratio greater than 1.290 were 28 times more likely to develop PE, with a sensitivity of 94.44% (95% CI: 74.24%–99.72%) and a specificity of 97.22% (95% CI: 85.83%–99.86%). The PPV was 50.82%, and the NPV was 49.71%. The LR was 34 (95% CI: 86.51%–99.87%), and the AUC was 0.9460 (95% CI: 0.8430–1.000), demonstrating excellent predictive strength (Figure 1C and Table 3).

To evaluate causal relationships among multiple variables simultaneously, multivariate analysis was employed (Table 4) to assess variables within the study's subject characteristics, with a significance threshold of *p* < 0.25.

**Table 4:** Multivariate linear regression models for the glyfn/plgf ratio PE

Variables	Estimate	SE	95% CI	*P*-Value
Maternal Age	−1.608	0.657	−2.952– −0.2645	0.02*
Body Weight before Pregnancy	−0.153	0.310	−0.7863–0.4813	0.63
Body Weight Increase during Pregnancy	−0.181	0.327	−0.8500–0.4883	0.58
Systolic Blood Pressure	−0.046	0.254	−0.5646–0.4722	0.86
Diastolic Blood Pressure	0.2555	0.3842	−0.5303–1.041	0.51
Constanta	77.02	41.02		

*Notes: Multivariate analysis was conducted using linear regression tests (95% CI) on numerical subject characteristic variables with a *P* value < 0.25

A *P* value < 0.05 is statistically significant

The GlyFn cutoff value of 34.31 ng/mL identified high-risk pregnancies to develop preeclampsia (PE) with an 83.33% sensitivity and 66.67% specificity (AUC = 0.8071). The PlGF cutoff value of 26.71 pg/mL identified high-risk pregnancies to develop preeclampsia (PE); yielded an 84.21% sensitivity and 97.14% specificity (AUC = 0.9045). The GlyFn/PlGF ratio cutoff of 1.290 provided the highest accuracy to identify high-risk pregnancies to develop preeclampsia (PE), with a sensitivity of 94.44%, specificity of 97.22%, and an AUC of 0.9460. These findings demonstrate the strong potential of these biomarkers for predicting preeclampsia in high-risk pregnancies.

 Multivariate linear regression models for the GlyFn/PlGF ratio in preeclampsia patients against other variables. Multivariate analysis was performed using linear regression tests (95% CI) on numerical subject characteristics with a *P* value < 0.25. Based on the analysis of numerical variables with a *P* value < 0.25, these were included as candidate multivariate variables. According to Table 4.6, maternal age is a factor that significantly influences the GlyFn/PlGF ratio in the PE group of patients with *P* = 0.02.

## Discussion

### Principal findings and results comparisons with previous studies

A GlyFn/PlGF ratio threshold of 1.290 yielded the highest diagnostic accuracy for identifying pregnancies at elevated risk of developing preeclampsia, achieving a sensitivity of 94.44%, specificity of 97.22%, and an AUC of 0.9460. These results highlight the significant predictive value of these biomarkers in assessing preeclampsia risk among high-risk pregnant women. These findings highlight the promising role of GlyFn/PlGF as a biomarker panel for early risk stratification in pregnancies susceptible to PE. This performance is comparable to, or even exceeds, that of currently established markers like sFlt-1/PlGF in certain populations.

Previous studies have consistently demonstrated that the GlyFn/PlGF ratio is a promising biomarker for predicting preeclampsia (PE), particularly in high-risk pregnancies. Elevated GlyFn levels and reduced PlGF levels have been associated with the onset and severity of PE. The combined ratio offers high diagnostic accuracy, with some studies reporting an area under the curve (AUC) of up to 0.94. Research by Moungmaithong et al. and Rasanen et al. showed that GlyFn levels correlate with PE severity and adverse outcomes such as preterm birth and low birth weight. The ratio has shown clinical utility in early gestational windows and across diverse populations, highlighting its potential as a reliable, non-invasive screening tool and a possible alternative or complement to existing biomarkers such as sFlt-1/PlGF [11, 15].

Importantly, GlyFn levels in our study were significantly higher in the PE group (mean 37.36 ± 10.45 ng/mL) compared to the non-PE group (27.29 ± 15.43 ng/mL, *P* = 0.0065), reinforcing its potential as a predictive tool. One mechanism that may explain why GlyFn increases in women with preeclampsia and/or diabetes mellitus is insulin resistance and inflammation. In pathological conditions, cFn levels rise in preeclampsia and inflammation. Changes in serum or plasma cellular Fn levels indicate alterations in the matrix and damage to the vessel walls. Based on its variants’ involvement, Fn can serve as a source of information for biomarkers, particularly in processes such as vascular remodeling disorders, inflammation, and hemostasis [12, 18–20].

In this study, GlyFn levels at 20–28 weeks were significantly higher in the preeclampsia (PE) group compared to the non-PE group (37.36 ± 10.45 ng/mL vs. 27.29 ± 15.43 ng/mL, *P* = 0.0065). This supports findings by Moungmaithong et al. (2023), which showed that GlyFn is a useful biomarker for PE screening, especially in the early gestational period. Elevated GlyFn levels are influenced by maternal age and can improve screening performance for PE. Their study further showed that elevated GlyFn levels at 11–13 weeks were significantly higher in PE pregnancies compared to non-PE pregnancies, and the addition of GlyFn improved the screening performance for premature PE and PE of any onset. Increased GlyFn levels have been reported in pregnant women of various ethnic backgrounds who develop PE after 20 weeks [16]. 32 Additionally, GlyFn was found to increase between 6–14 weeks Previous studies also reported higher GlyFn levels in PE pregnancies, with satisfactory predictive results (AUC 0.94) in high-risk cohorts, including studies in Southeast Asia. Additionally, GlyFn has been linked to adverse pregnancy outcomes such as preterm birth, low birth weight, and high blood pressure, demonstrating its clinical utility in identifying high-risk women for PE [12, 17]. Wang et al.‘s research found higher GlyFn levels in the PE group, though the difference was not statistically significant, possibly due to study design factors. Rasanen et al. (2020) showed that GlyFn levels correlated with PE severity and adverse outcomes like high blood pressure, preterm delivery, and low birth weight. Their study found significant increases in GlyFn levels in women with PE, especially those with severe cases, while no significant changes were seen in normotensive women. These findings highlight GlyFn’s clinical value in predicting and diagnosing preeclampsia and identifying high-risk women [12, 17–20].

In comparison, PlGF is a trophoblast-derived angiogenic factor whose downregulation in PE has been widely documented. Our results affirm that PlGF levels were markedly reduced in PE cases, and the combination with GlyFn strengthens diagnostic accuracy. Changes in angiogenic factors, particularly PlGF, strongly correlate with preeclampsia, often preceding its clinical onset by weeks. Reduced levels of PlGF, along with other angiogenic factors like VEGF, are observed in women with preeclampsia, and these anti-angiogenic properties are associated with endothelial dysfunction. Lower PlGF concentrations, detectable before the clinical diagnosis of preeclampsia, are also linked to the severity of the condition, with early-onset or severe cases showing the most significant reductions in PlGF levels [12, 18–20]. Several studies have reported that women who later develop preeclampsia exhibit significantly lower PlGF levels in the first trimester compared to those with normal pregnancies. PlGF has detection rates of 55% for early-onset and 33% for late-onset preeclampsia at a 10% false-positive rate. A systematic review found PlGF to outperform other biomarkers for early-onset preeclampsia with a 56% detection rate at a 9% false-positive rate [12, 18–20]. Despite variations in diagnostic accuracy across studies, PlGF levels are consistently lower in preeclamptic pregnancies, suggesting its utility as both a biomarker and mediator of endothelial dysfunction in preeclampsia [12, 18–20].

### Clinical implication and further direction

Given these results, the GlyFn/PlGF ratio shows promise as an alternative or complementary approach to sFlt-1/PlGF testing. While sFlt-1/PlGF is already integrated into clinical practice in some countries, its high cost and limited availability in low-resource settings present significant barriers [19]. Future research should focus on larger, diverse populations to validate these findings and explore the ratio’s application in clinical settings. Integration of this biomarker into routine prenatal care may enhance early detection and management of preeclampsia, head-to-head comparisons with sFlt-1/PlGF in diverse populations, and cost-effectiveness analyses to assess clinical utility in routine obstetric care. Additionally, investigating the molecular mechanisms regulating GlyFn expression could further refine its application and enhance our understanding of PE pathogenesis.

### Strength and limitations

Given the lack of local data from Indonesia on this topic, this study represents an effort to present observations on the Indonesian population. The main advantage of this research is that, to the best of our knowledge, it is the first study to investigate the biomarker profile with the GlyFn/PlGF ratio in high-risk pregnant women in Indonesia. Worldwide, there is only one study by Wang et al. that assesses several biomarkers, including the GlyFn/PlGF ratio. This study found a cut-off value of 1.288, supported by other significant values such as sensitivity, specificity, PPV, NPV, and AUC, all demonstrating strong and meaningful significance. Given the limited global and local data, this research represents an important observation for the Indonesian population.

However, there are several limitations that need to be considered. First, as a preliminary study, our research is limited to a small sample size, necessitating further studies with a larger population that can better represent the Indonesian population. Second, since samples were taken from a regional area and laboratory analyses were conducted in a major city, the impact of sample transportation needs to be considered. Third, other laboratory data were not analyzed in this study to minimize bias. Nonetheless, these findings still require confirmation with other laboratory profiles and metabolites.

## Conclusion

The GlyFn/PlGF ratio is a promising biomarker for predicting preeclampsia in high-risk pregnancies. Its application can improve early detection and clinical decision-making, contributing to better maternal and fetal outcomes. Further studies are warranted to confirm its diagnostic utility and optimize its integration into clinical practice

## Data Availability

The datasets used and/or analysed during the current study are available from the corresponding author on reasonable request.

## Abbreviations

BMI: Body mass index
DBP: Diastolic blood pressure
EVT: Extravillous trophoblast
Flt-1: Fms-like tyrosine kinase-1
Fn: Fibronectin
GlyFn: Glycosylated Fibronectin
IL: Interleukin
PE: Preeclampsia
pFn: Plasma fibronectin
PIGF: Placental growth factor
RAAS: Renin-angiotensin-aldosteron
ROC: Receiving operator characteristics
SBP: Systolic blood pressure
sFlt-1: Soluble Flt-1
SLE: Systemic Lupus Erythematosus
sENG: Soluble Endoglin
TNF-α: Tumor necrosis factor-α
VEGF: Vascular endothelial growth factor
